# Kangtaizhi Granule Alleviated Nonalcoholic Fatty Liver Disease in High-Fat Diet-Fed Rats and HepG2 Cells via AMPK/mTOR Signaling Pathway

**DOI:** 10.1155/2020/3413186

**Published:** 2020-08-20

**Authors:** Jiaxin Zhang, Haixia Du, Menglan Shen, Zhengqi Zhao, Xinmiao Ye

**Affiliations:** ^1^College of Basic Medical Science, Zhejiang Chinese Medical University, Hangzhou, 310053 Zhejiang, China; ^2^College of Pharmaceutical Science, Zhejiang Chinese Medical University, Hangzhou, 310053 Zhejiang, China

## Abstract

Kangtaizhi granule (KTZG) is a Chinese medicine compound prescription and has been proven to be effective in nonalcoholic fatty liver disease (NAFLD) treatment clinically. However, the underlying mechanisms under this efficacy are rather elusive. In the present study, network pharmacology and HPLC analysis were performed to identify the chemicals of KTZG and related target pathways for NAFLD treatment. Network pharmacology screened 42 compounds and 79 related targets related to NAFLD; HPLC analysis also confirmed six compounds in KTZG. Further experiments were also performed. In an *in vivo* study, SD rats were randomly divided into five groups: control (rats fed with normal diet), NAFLD (rats fed with high-fat diet), and KTZG 0.75, 1.5, and 3 groups (NAFLD rats treated with KTZG 0.75, 1.5, and 3 g/kg, respectively). Serum lipids were biochemically determined; hepatic steatosis and lipid accumulation were evaluated with HE and oil red O staining. In an *in vitro* study, HepG2 cells were incubated with 1 mM FFA to induce lipid accumulation with or without KTZG treatment. MTT assay, intracellular TG level, oil red O staining, and glucose uptake in cells were detected. Western blotting and immunohistochemical and immunofluorescence staining were also performed to determine the expression of lipid-related genes PPAR-*γ*, SREBP-1, p-AKT, FAS, and SIRT1 and genes in the AMPK/mTOR signaling pathway. In high-fat diet-fed rats, KTZG treatment significantly improved liver organ index and serum lipid contents of TG, TC, LDL-C, HDL-C, ALT, and AST significantly; HE and oil red O staining also showed that KTZG alleviated hepatic steatosis and liver lipid accumulation. In FFA-treated HepG2 cells, KTZG treatment decreased the intracellular TG levels, lipid accumulation, and attenuated glucose uptake significantly. More importantly, lipid-related genes PPAR-*γ*, SREBP-1, p-AKT, FAS, and SIRT1 expressions were ameliorated with KTZG treatment in high-fat diet-fed rats and FFA-induced HepG2 cells. The p-AMPK and p-mTOR expressions in the AMPK/mTOR signaling pathway were also modified with KTZG treatment in high-fat diet-fed rats and HepG2 cells. These results indicated that KTZG effectively ameliorated lipid accumulation and hepatic steatosis to prevent NAFLD in high-fat diet-fed rats and FFA-induced HepG2 cells, and this effect was associated with the AMPK/mTOR signaling pathway. Our results suggested that KTZG might be a potential therapeutic agent for the prevention of NAFLD.

## 1. Introduction

Nonalcoholic fatty liver disease (NAFLD) is characterized by the excessive accumulation of hepatic lipid and hepatic steatosis in the absence of alcoholism [1]. It is emerging as the most common cause of liver diseases, with a global prevalence of 25% approximately. More importantly, NAFLD is considered to be a dangerous complication of nonalcoholic steatohepatitis (NASH), advanced fibrosis, cirrhosis, and hepatocellular carcinoma, with evidence of NAFLD at markedly increased risk of adverse outcomes, including liver-specific morbidity and mortality [2]. Recently, increases in high-fat diet (HFD) intake and thus induced obesity have demonstrated parallelism with a global increase in NAFLD. Furthermore, studies evidence that NAFLD contributed to the characteristics of a series of diseases, such as metabolic syndromes, like type II diabetes mellitus, hypertension, and cardiovascular events, making NAFLD a major public health concern worldwide [3, 4].

Although the presence of steatosis is a requisite and typical for NAFLD, the development of NAFLD is a complex process that is affected by numerous mechanisms including genetic, metabolic, lifestyle, and gut microbiome [5]. They lead to increased metabolic substrate (mainly lipids and carbohydrates) delivery to the liver and increased visceral adipose tissue, featured with the excessive accumulation of free fatty acids (FFA), triglycerides (TG), and proinflammatory mediators. These changes alter lipid and glucose metabolism, produce insulin resistance, and create a proinflammatory milieu that induce oxidative stress and modify cell-cell crosstalk, triggering cell injury, apoptosis, or cell death [6]. Variable activation of these processes and the consequent metabolic response determines the development of disease phenotype and its progression to fibrosis and cirrhosis. Lifestyle intervention is recommended for the majority of NAFLD patients, and its benefit is also witnessed by a wide range of clinical studies [7, 8]. However, confined to the difficulties of long-term exercise, pharmaceutical treatment aimed at alleviating hepatic steatosis or protecting the liver from additional injury is necessary.

To expand the range of pharmaceutical options for NAFLD treatment, recent studies also focus on identifying active agents or herbal extracts that can ameliorate NAFLD. With a multi-ingredient and multipath pharmacological action, traditional Chinese medicine (TCM) is compatible with the complex pathogenesis of NAFLD, which can be used to mitigate NAFLD and associated diseases [9]. Kangtaizhi granule (KTZG) is a Chinese medicine compound prescription that is composed of eight kinds of TCM, Radix Puerariae, Rhizoma Dioscoreae, Sophora japonica oyster, mulberry leaves, Polygonatum odoratum, mulberry, and papaya. Some of these eight TCM are partly consumed as a part of the human diet with abundant bioactivities. Our previous study demonstrated that KTZG has the efficacy to protect life from NAFLD, with the ability to ameliorate abnormal liver function and lipid metabolism [10]. Although the effect of KTZG in the clinic has been confirmed, the clear mechanisms under this efficacy are rather elusive. In this study, with network pharmacology and experiment validation in high-fat diet rats and FFA-treated HepG2 cells, we aimed to provide basic data for a better understanding of the pharmacological effects and potential mechanisms of KTZG in preventing of NAFLD.

## 2. Materials and Methods

### 2.1. KTZG Bioactive Compounds and NAFLD-Related Target Screening

For the eight herbs that make up KTZG, the Traditional Chinese Medicine Systems Pharmacology (TCMSP) database, TCM-Mesh database, and Traditional Chinese Medicine Integrative Database (TCMID) database were used to obtain the related chemical information of these eight herbs, including the bioactive compound name; molecular weight; structural formula; absorption, distribution, metabolism, and elimination (ADME) parameters; and CAS ID. Based on the screened compounds in KTZG, compound-related targets were predicted using the SwissTargetPrediction and STITCH online databases. Canonical SMILES of each compound was searched from PubChem and submitted to these two databases, then predicted targets were obtained. The keyword “NAFLD” was inputted in the following databases including the Therapeutic Target Database (TTD), DrugBank, Online Mendelian Inheritance in Man (OMIM), and DisGeNET to screen the disease-related targets. Then, the coexistent targets between compounds and disease were identified as KTZG-related targets for NAFLD treatment.

### 2.2. Network Construction and Analysis

Based on the identified compounds and predicted targets of KTZG, the interaction network between compounds and targets was visualized with Cytoscape 3.2.1 software. The protein-protein interactions of the targets were also analyzed with the STRING online tool and visualized with Cytoscape 3.2.1 software. The protein's topological attribute was analyzed with the software plugin tool “Network analyzer”.

### 2.3. KEGG Pathway Enrichment Analysis

For the predicted targets, the underlying biological information was also explored. These target-enriched KEGG pathway networks were analyzed with the Cytoscape 3.2.1 software plugin tool ClueGO; *P* value of the KEGG pathway term less than 0.05 was set up as the criterion.

### 2.4. High-Performance Liquid Chromatography (HPLC) Analysis of KTZG

HPLC analysis of KTZG was performed on a Waters e2695 HPLC system equipped with a 2998 PDA detector. An Agilent ZORBAX SB-C18 column (4.6 mm × 150 mm, 5 *μ*m) was used for the chromatographic separation, and solvent A (acetonitrile) and solvent B (0.01% glacial acetic acid in distilled water) composed the solvent system. The chromatographic separation was conducted according to the following solvent gradient: 0-30 min, 10%-15% A; 30-60 min, 15%-35% A; and 60-70 min, 35%-90% A. the injection volume was 10 *μ*L, flow rate was 0.8 mL/min, detection wavelength was 254 nm, and column temperature was 30°C [11].

### 2.5. Animal Diets and Tissue Sample Preparation

4-week male SD rats were purchased from the Shanghai SLAC Laboratory Animal Co. Ltd., China, and maintained under specific pathogen-free conditions at 22~25°C temperature, 60% humidity, 12 h dark-light cycle, and free access to water and normal diet. After a week of acclimatization, the rats were randomly divided into five groups (*n* = 12 per group): control group: rats were fed with normal diet; NAFLD group: rats were fed with HFD; KTZG 0.75 group: rats were fed HFD and KTZG 0.75 mg/kg/d, i.g.; KTZG 1.5 group: rats were fed with HFD and KTZG 1.5 mg/kg/d, i.g.; and KTZG 3 group: rats were fed with HFD and KTZG 3.0 mg/kg/d, i.g. Rats were fed with normal or HFD for 10 weeks; at the same time, KTZG was administrated from the week of 5 to 10 for a total of 6 weeks. The same volume of saline was given to the control and NAFLD groups. After last administration and overnight fasting, body weight was recorded, and all rats were euthanized using CO_2_. Blood was collected from a cardiac puncture and centrifugated at 3000 rpm for 15 min to obtain the serum samples. Liver tissues were also removed and weighted and then stored immediately at -80°C for further analysis. All experiments were performed with the approval from the Zhejiang Chinese Medical University using guidelines for the care and use of laboratory animals.

### 2.6. Biochemical Analysis

Serum content of total TG, total cholesterol (TC), total bilirubin, high-density lipoprotein (HDL), low-density lipoprotein (LDL), alanine aminotransferase (ALT), and aspartate aminotransferase (AST) was detected using an automatic analyzer (Roche, Basel, Switzerland) according to the manufacturer's introduction.

### 2.7. Liver Histopathological Examination

The liver tissues were fixed in 10% paraformaldehyde for 48 h and embedded in paraffin, then the tissues were cut into 3~5 *μ*m sections and stained with hematoxylin and eosin (HE). All the specimens were examined under a light microscope (Olympus, Tokyo, Japan). The tissue sections were also stained with oil red O solution to assess the lipid droplet accumulation in liver tissues. The specimens were examined under a light microscope, and the oil red O-positive areas were quantified using Image-Pro Plus 6.0 software (IPP, Media Cybernetics, Inc., USA).

### 2.8. Immunohistochemical Assay

Liver tissue sections were incubated with the primary antibody at 4°C overnight, then washed with PBS for three times and further incubated with the horseradish peroxidase anti-rabbit secondary antibody for 15 min at room temperature. Then, the sections were washed with PBS for 3 times and stained with DBA solution. After terminating the reaction with water, the sections were further counterstained with hematoxylin; the tissue sections in each group were observed under a microscope.

### 2.9. Cell Culture and Induction of Hepatic Steatosis

The HepG2 hepatocyte cell line (iCell Bioscience Inc., China) was cultured in Dulbecco's Modified Eagle's Medium (DMEM, Hyclone) supplemented with 10% FBS and penicillin/streptomycin mixture at 37°C in a 5% CO_2_ atmosphere. For induction of hepatic steatosis, HepG2 cells were incubated with 1 mM of FFA (oleic acid and palmitic acid, 2 : 1) in serum-free medium containing 1% fat-free bovine serum albumin (BSA) to stimulate lipid accumulation for 24 h. Serum samples containing KTZG were obtained from the KTZG 0.75, 1.5, 3 mg/k group normal diet-fed SD rats and named the L-KTZG, M-KTZG, and H-KTZG groups, respectively. Then, the cells were further incubated in DMEM-contained serum (20 *μ*L) containing with KTAG for another 24 h. The control group and FFA group HepG2 cells were incubated in DMEM-contained normal serum (20 *μ*L) without KTZG.

### 2.10. MTT Assay

HepG2 cells (1 × 10^4^ cells/well) were seeded in a 96-well plate and allowed to adhere to the wells. Then, cells were incubated in serum-free DEME containing 1 mM of FFA, with or without KTZG treatment at different doses for 24 h. Then, MTT reagent (BBI Life Sciences) was added for another coincubation of 1 h. After that, the optic density (OD) value was measured at 590 nm using a CMax Plus ELISA reader (MD, USA).

### 2.11. Intracellular TG Level Assay

HepG2 cells (1 × 10^4^ cells/well) were seeded in 6-well plates and allowed to adhere to the wells. Then, cells were incubated in DMEM containing 1 mM of FFA, with KTZG treatment at different doses for 24 h. Then, intracellular TG levels were detected with the TG assay kit (Cayman Chemical, USA) according to the manufacturer's instructions.

### 2.12. Oil Red O Staining

Oil red O staining was also performed to evaluate the intracellular lipid accumulation. Briefly, the prepared HepG2 cells were fixed with 10% formalin in PBS for 1 h and then stained with oil red O solution for 30 min at room temperature. Then, cells were washed with distilled water, and then the stained lipid droplets within cells were observed under a light microscope. The lipid accumulation was quantified through the dissolving of stained lipid droplets with isopropanol, and the absorbance was measured at 490 nm. Control cells were treated with 1% BSA only.

### 2.13. Glucose Uptake Assay

Glucose uptake was detected using a fluorescence assay. HepG2 cells (1 × 10^4^ cells/well) were seeded in a 6-well plate and treated as described above. Then, the medium was removed, and 100 *μ*mol/Lof 2-NBDG was added and coincubated in serum-free DMEM medium at 37°C for 30 min. Then, the relative fluorescence images were observed under a Ts2-FC fluorescence microscope (Nikon, Japan).

### 2.14. Immunofluorescence Staining

HepG2 cells were prepared as described above and fixed with 10% formalin in PBS for 1 h. Then, the fixed cells were washed with PBS for three times, permeabilized in 0.5% Triton X-100, and then blocked with 5% normal goat serum. After incubation with primary p-AKT (ab23875, 1 : 250, Abcam, USA), p-mTOR (ab131538, 1 : 250, Abcam, USA), and secondary antibody IgG H&L (Alexa Fluor® 594) (ab150076, 1 : 250, Abcam, USA), cell nuclei were stained with DAPI (Beyotime Institute of Biotechnology, China). Then, the relative fluorescence images were obtained using a Ts2-FC fluorescence microscope (Nikon, Japan).

### 2.15. Western Blot Assay of Liver Tissues and HepG2 Cells

Liver tissues or HepG2 cells were lysed with RIPA buffer (Beyotime Institute of Biotechnology, China), and the protein concentrations were measured by the BCA method using a BCA protein assay kit (Beyotime Institute of Biotechnology, China). Then, equal amounts of protein samples (30 *μ*g) were loaded onto 10% SDS-polyacrylamide gel and electrophoretically transferred to polyvinylidene difluoride (PVDF) membranes. The membranes were blocked with the blocking solution and incubated with the primary antibodies against PPAR-*γ*, ab59256, 1 : 1000; SREBP-1, ab28481, 1 : 5000; p-AKT, ab8805, 1 : 500; FAS, ab82419, 1 : 1000; SIRT1, ab12193, 1 : 2000; p-AMPK, ab23875, 1 : 1000; AMPK, ab80039,1 : 1000; p-mTOR, ab131538, 1 : 1000; mTOR, ab32028, 1 : 5000; and GLUT2, ab192599, 1 : 5000 (Abcam, USA) overnight at 4°C. After that, the membranes were washed with TBST for three times and further incubated with horseradish peroxidase- (HRP-) conjugated secondary antibodies (ab20272, 1 : 5000). The blots were visualized using Millipore's enhanced chemiluminescence and quantified using Image-Pro Plus 6.0 software (IPP, Media Cybernetics, Inc., USA).

### 2.16. Statistical Analysis

Data were expressed as mean ± SD and analyzed with SPSS software; statistical significance was determined by Student's *t*-test for comparisons between two groups. Differences were considered to be significant when *P* < 0.05.

## 3. Results

### 3.1. TKZG-Compound-Target Network

**Figure 1:**
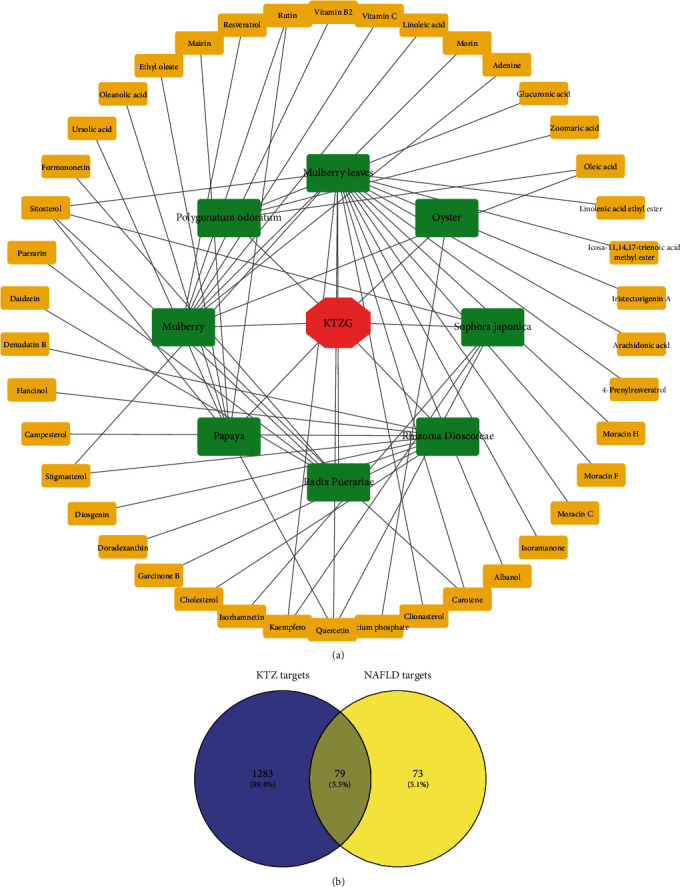
KTZG-bioactive compounds and NAFLD-related target screening. (a) KTZG-bioactive compound network. (b) Screening of the protein-targeted KTZG for NAFLD treatment. KTZG: Kangtaizhi granule; NAFLD: nonalcoholic fatty liver disease.

**Figure 2:**
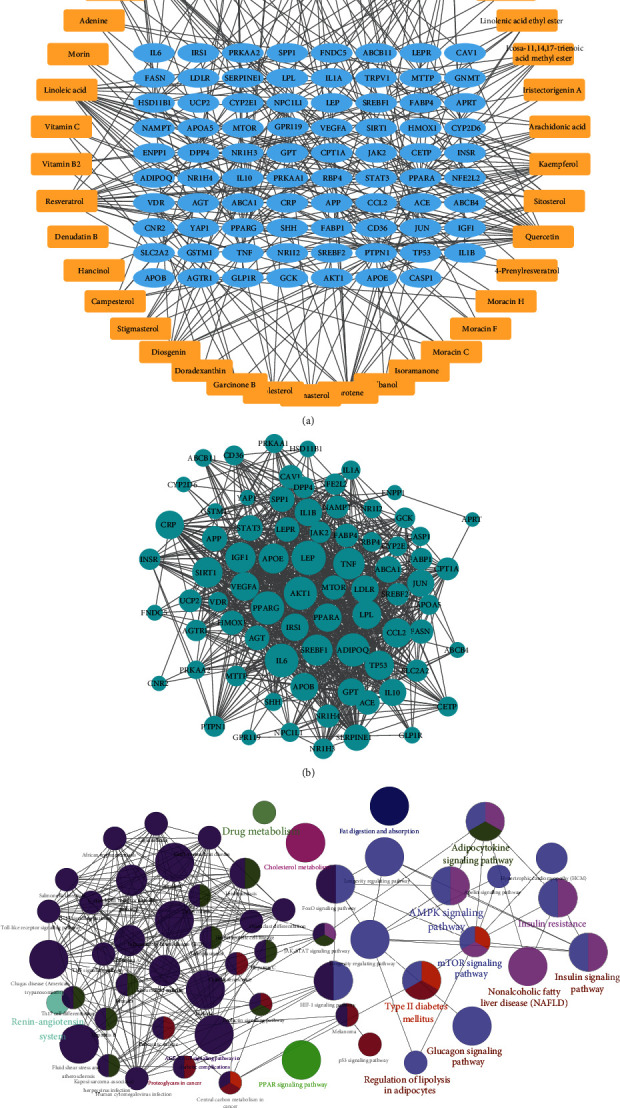
Network pharmacology analysis of the KTZG for NAFLD treatment. (a) Compound-target network of KTZG. The network included 42 compounds and 79 proteins, forming 296 edges. (b) PPI network of the identified targets. Node size was positively associated with node degree. (c) KEGG pathway analysis of the targets.

TKZG was prepared by eight TCM. Based on TCM-related databases, the chemical constituents in these eight TCM were screened (Figure 1(a)). In addition, 1362 proteins that target these compounds were predicted, and 152 protein targets related to NAFLD were screened. As a result, 79 coexisted targets were identified as TKZG's targets for NAFLD treatment (Figure 1(b)). The compound-target network is also shown in Figure 2(a), including 42 compounds and 79 proteins, forming 296 edges.

### 3.2. Hub Target Identification

The PPI network was constructed to analyze the interactions of these identified targets (Figure 2(b)). 77 targets were mapped into the network with 1015 interaction edges, forming a complex interaction network. The node size was positively correlated to the node degree; some proteins were specified with a high degree, including PPARG (degree = 54), ADIPOQ (degree = 54), AKT1 (degree = 54), TNF (degree = 49), SREBF1 (degree = 48), and SIRT1 (degree = 46).

### 3.3. KEGG Pathway Analysis

79 target-enriched KEGG pathways were also analyzed; the KEGG pathway network is also presented in Figure 2(c). The network was composed of 84 terms of pathways, forming a tight pathway interaction network. Some pathways that the identified targets enriched were directly related to the disease NAFLD, including the NAFLD pathway, type II diabetes mellitus, insulin resistance, insulin signaling pathway, glucagon signaling pathway, regulation of lipolysis in adipocytes, and other signaling pathways like the AMPK and mTOR signaling pathways. These signaling pathways directly interacted with NAFLD-associated pathways and marked with similar color cluster.

### 3.4. HPLC Chemical Fingerprint of KTZG

**Figure 3:**
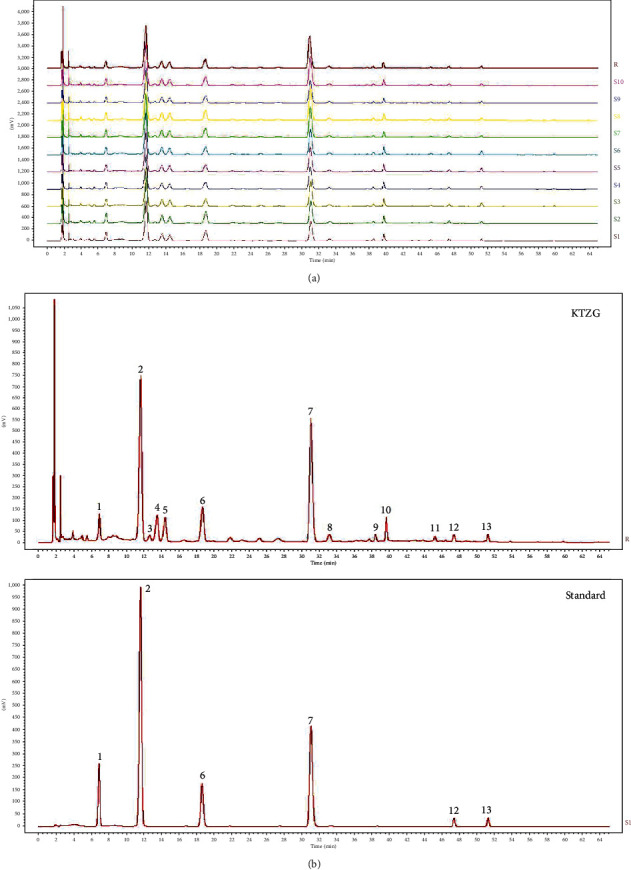
HPLC chemical fingerprint of KTZG. (a) The reproducible HPLC chromatograms of HTT from 10 batches. (b) HPLC chemical fingerprint of KTZG with 6 peaks determined by comparing retention time with the standards: 3′-hydroxy puerarin (no. 1), puerarin (no. 2), daidzin (no. 6), rutin (no. 7), daidzein (no. 12), and quercetin (no. 13).

Ten batches of KTZG were prepared, and the typical chromatograms from these 10 batches with good quality control are shown in Figure 3(a) by HPLC analysis. Thirteen peaks were identified on the HPLC chemical fingerprint of KTZG (Figure 3(b)). Among these peaks, six peaks, including 3′-hydroxy puerarin (no. 1), puerarin (no. 2), daidzin (no. 6), rutin (no. 7), daidzein (no. 12), and quercetin (no. 13), were identified by referring to the corresponding standards.

### 3.5. KTZG Improved Liver Organ Index and Serum Lipid Profile

**Figure 4:**
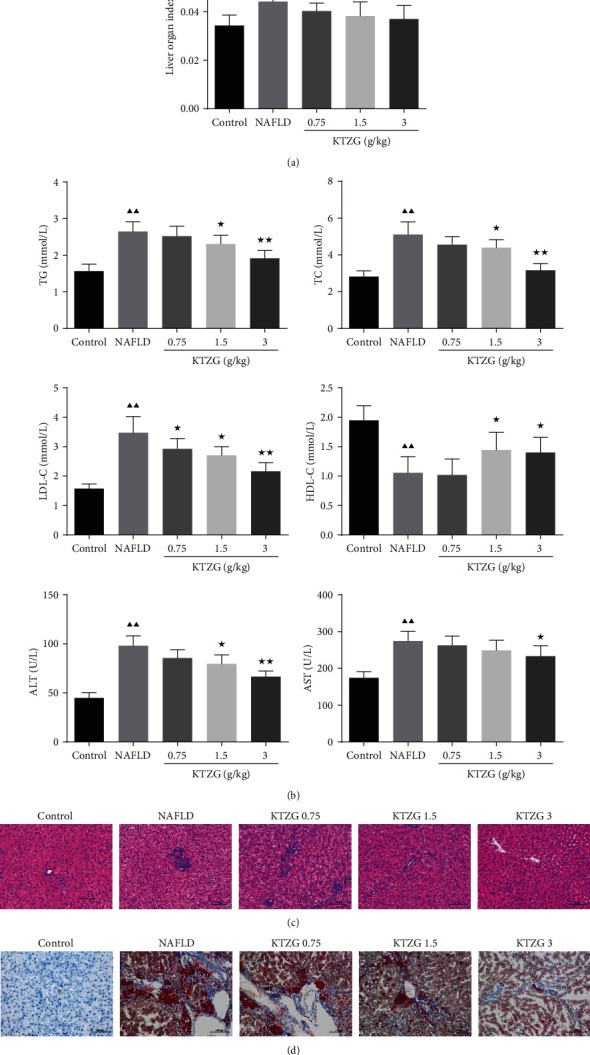
Effect of KTZG on lipid accumulation and hepatic steatosis in HFD-fed rats. (a) Liver organ index of the HFD-fed rat liver tissues treated with KTZG. (b) Serum content of TG, TC, LDL-c, HDL-c, ALT, and AST. (c) HE staining of liver tissues (magnification ×200). (d) Oil red O staining images of the HFD-fed rat liver tissues treated with KTZG (magnification ×200). HFD: high-fat diet. Compared to control group, ^▲^*P* < 0.05, ^▲▲^*P* < 0.01; compared to NAFLD group, ^★^*P* < 0.05, ^★★^*P* < 0.01.

In the NAFLD model rat with HFD, the liver organ index was significantly increased compared to that in the normally fed control group rat (*P* < 0.05) (Figure 4(a)). But for model rats with KTZG doses reaching 1.5 g/kg and 3 g/kg, the organ index was significantly decreased (*P* < 0.05 and *P* < 0.01, respectively). Compared to the control group, serum contents including TG, TC, and LDL-c in the model group were significantly increased (*P* < 0.05) (Figure 4(b)). But KTZG treatment inhibited these increased lipid profiles in the serum, especially in the 1.5 g/kg and 3 g/kg dosage groups with statistical significance. For HDL-c, the levels in these groups were correspondingly opposite. In addition, serum AST and serum ALT in the NAFLD group were significantly higher than those in the control group (*P* < 0.01), and the serum AST and ALT levels in the KTZG group were significantly decreased than those in the NAFLD group (*P* < 0.05).

### 3.6. KTZG Alleviated Hepatic Steatosis and Liver Lipid Accumulation in NAFLD Rats

HE staining showed that compared to the control group with normal liver morphology, NAFLD group liver tissues showed significantly hepatic steatosis, vacuolation, hepatocellular hypertrophy, and lipid droplet accumulation (Figure 4(c)), whereas the degree of hepatic steatosis and the size of the lipid droplets were alleviated in the liver of the rats with KTZG treatment. Oil red O results also evidenced that there were significant lipid droplets and lipid accumulation in liver tissues of NAFLD rats, and KTZG treatment reduced the HFD-induced lipid accumulation in liver tissues (Figure 4(d)).

### 3.7. Effect of KTZG on Expression of Lipid-Related Genes and AMPK/mTOR Signaling in Liver Tissues

**Figure 5:**
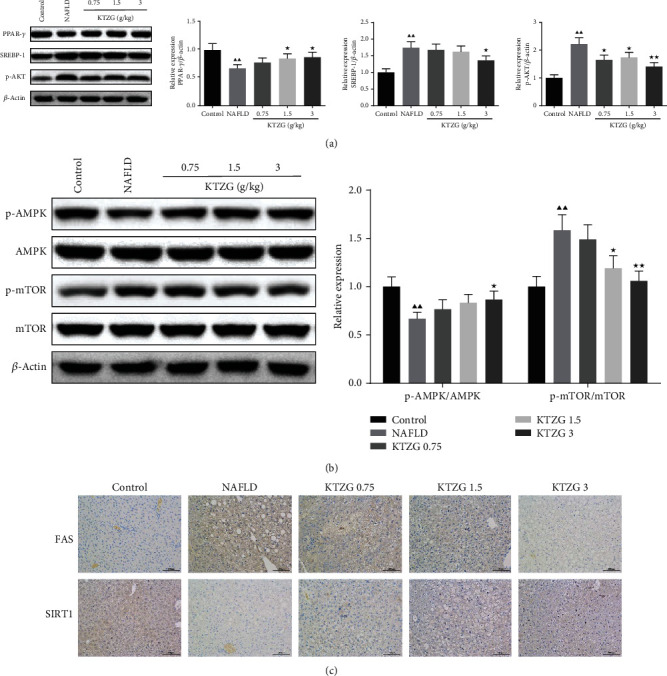
Effect of KTZG on PPAR-*γ*, SREBP-1, p-AKT, FAS, SIRT1, and AMPK/mTOR signaling pathway in HFD fed rats. (a) Western blotting for PPAR-*γ*, SREBP-1, and p-AKT in HFD-fed liver tissues treated with KTZG. (b) Western blotting for p-AMPK, AMPK, p-mTOR, and mTOR in HFD-fed liver tissues treated with KTZG. (c) Immunohistochemical of FAS and SIRT1 in liver tissues (magnification ×200). Compared to control group, ^▲^*P* < 0.05, ^▲▲^*P* < 0.01; compared to NAFLD group, ^★^*P* < 0.05, ^★★^*P* < 0.01.

Expressions of the lipid-related genes identified from network pharmacology and the proteins in AMPK/mTOR signaling were detected. Western blot results in Figure 5(a) showed that the protein expressions of PPAR-*γ* were significantly decreased, and SREBP-1 and p-AKT were significantly increased in the NAFLD group compared to the control group (*P* < 0.01). KTZG treatment significantly increased the expression of PPAR-*γ* in the 1.5 and 3 g/kg groups (*P* < 0.05) and decreased the expression of SREBP-1 in the 3.0 g/kg group (*P* < 0.05) and p-AKT in the 0.75, 1.5, and 3 g/kg groups (*P* < 0.05, *P* < 0.05, and *P* < 0.01, respectively). Compared to the control group, the p-AMPK expression was significantly decreased in the NAFLD group, and KTZG treatment increased the expression significantly in the 3 g/kg group (*P* < 0.05); p-mTOR expression was significantly increased in the NAFLD group, and KTZG treatment increased the expression significantly in the 1.5 g/kg and 3 g/kg groups (*P* < 0.05 and *P* < 0.01, respectively) (Figure 5(b)). Immunohistochemical results also showed that compared to the control group, the expression of FAS was increased in the NFALD group but increased with KTZG treatment and the Sirt1 expression was decreased in the NFALD group but increased in the KTZG groups (Figure 5(c)).

### 3.8. Cell Viability of HepG2 Cells Treated with KTZG

**Figure 6:**
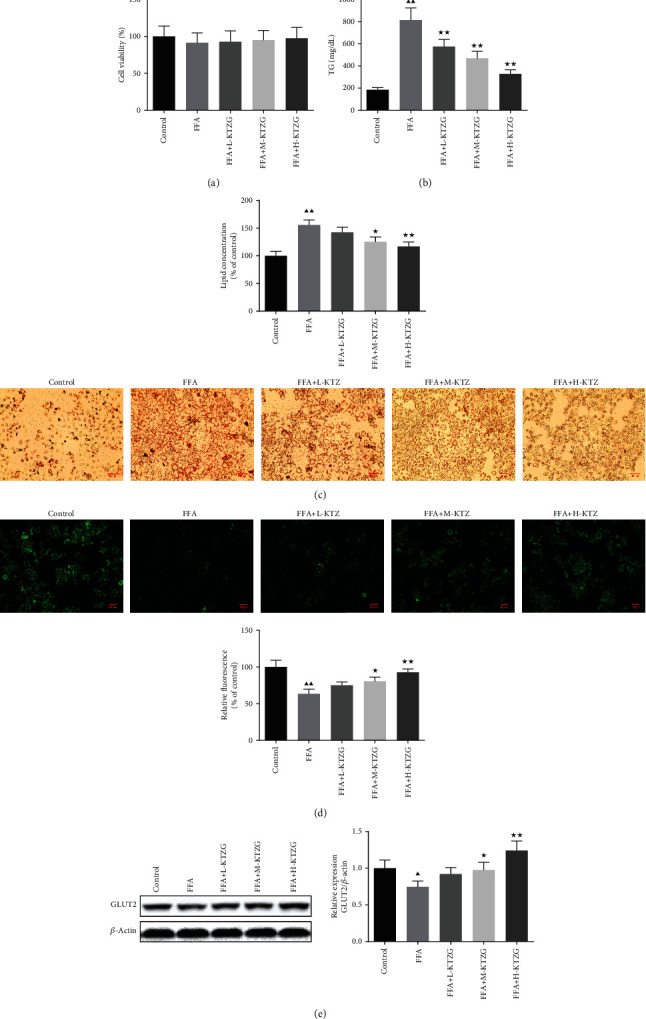
Effect of KTZG on lipid accumulation and hepatic steatosis in FFA-induced HepG2 cells. (a) MTT assay of the HepG2 cells. HepG2 cells were incubated in KZTG with FFA for 24 h. FFA: free fatty acids. (b) Intracellular TG levels in HepG2 cells treated with KTZG and FFA. (c) Oil red O staining images of HepG2 cells treated with KTZG and FFA (magnification ×200). (d) Effect of KTZG on the glucose uptake in FFA-induced HepG2 cells. (e) Western blotting for GLUT2 in HFD-fed liver tissues treated with KTZG. Compared to control group, ^▲^*P* < 0.05, ^▲▲^*P* < 0.01; compared to FFA group, ^★^*P* < 0.05, ^★★^*P* < 0.01.

The MTT assay was used to evaluate the effects of KTZG in different concentrations on HepG2 cell viability. As the result indicated, KTZG treatment at the tested concentrations did not exhibit significant toxicity in cell viability (Figure 6(a)).

### 3.9. KTZG Decreased Intracellular Lipid Accumulation in FFA-Induced HepG2 Cells

Significantly high intracellular TG level was induced by an exposure to FFA 1 mM for 24 h of HepG2 cells (Figure 6(b)), whereas in KTZG-treated cells, TG accumulation showed a significant decrease compared to that in FFA-only treated cells (*P* < 0.01). Oil red O staining showed that there was significant lipid droplet accumulation in FFA-treated HepG2 cells (*P* < 0.01), but for the middle and high concentrations of KTZG along with FFA treatment, the lipid accumulation was significantly decreased compared to those for the FFA-only treated cells (*P* < 0.05 and *P* < 0.01, respectively) (Figure 6(c)).

### 3.10. KTZG Attenuated Glucose Uptake in FFA-Induced HepG2 Cells

To determine the effect of KTZG on glucose uptake in HepG2 cells, the 2-NBDG glucose uptake assay was performed in HepG2 cells (Figure 6(d)). The result showed that the intensity of the glucose-labeled fluorescence was significantly decreased in response to the FFA treatment (*P* < 0.01). However, HepG2 cells treated with KTZG improved the glucose uptake, with higher fluorescence intensity, compared to cells treated with FFA only. Protein expression of GLUT2 also showed that the expression of GLUT2 was decreased in FFA-treated cells but substantially increased in response to KTZG.

### 3.11. Effect of KTZG on Expression of Lipid-Related Genes in FFA-Induced HepG2 Cells

**Figure 7:**
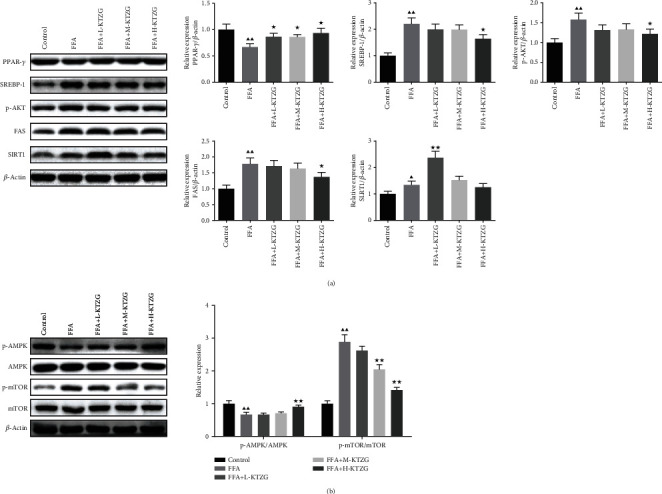
Effect of KTZG on PPAR-*γ*, SREBP-1, p-AKT, FAS, SIRT1, and AMPK/mTOR signaling pathway in FFA-induced HepG2 cells. (a) Western blotting for PPAR-*γ*, SREBP-1, and p-AKT. (b) Western blotting for p-AMPK, AMPK, p-mTOR, and mTOR. Compared to control group, ^▲^*P* < 0.05, ^▲▲^*P* < 0.01; compared to FFA group, ^★^*P* < 0.05, ^★★^*P* < 0.01.

Protein expressions of the lipid-related genes PPAR-*γ*, SREBP-1, p-AKT, FAS, and SIRT1 were detected in FFA-induced HepG2 cells. Western blot results in Figure 7(a) showed that the protein expressions of PPAR-*γ* were significantly decreased and SREBP-1, p-AKT, FAS, and SIRT1 were significantly increased in FFA-induced HepG2 cells compared to untreated cells (*P* < 0.01). In KTZG-treated HepG2 cells, significantly increased expressions of PPAR-*γ* were observed in KTZG low- to high-treated groups (*P* < 0.05); decreased expressions of SREBP-1, p-AKT, and FAS were observed in the KTZG high-treated group (*P* < 0.05).

### 3.12. KTZG Ameliorates Lipid Accumulation and Hepatic Steatosis in FFA-Induced HepG2 Cells via the Regulation of AMPK/mTOR-Dependent Signaling

**Figure 8:**
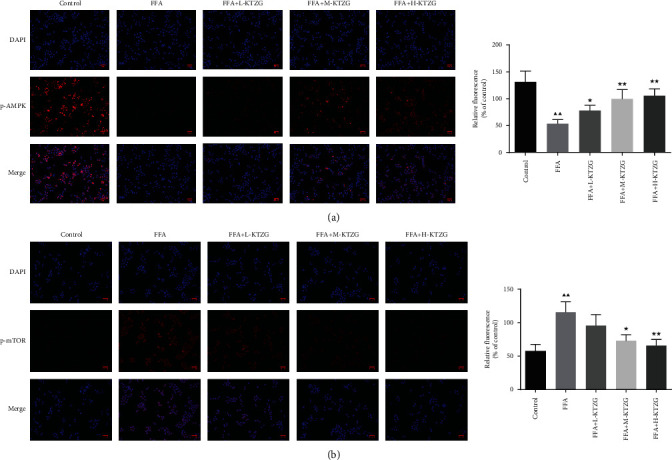
Immunofluorescence staining of p-AMPK (a) and p-mTOR (b) in FFA-induced HepG2 cells (magnification ×200). Compared to control group, ^▲^*P* < 0.05, ^▲▲^*P* < 0.01; compared to FFA group, ^★^*P* < 0.05, ^★★^*P* < 0.01.

**Figure 9:**
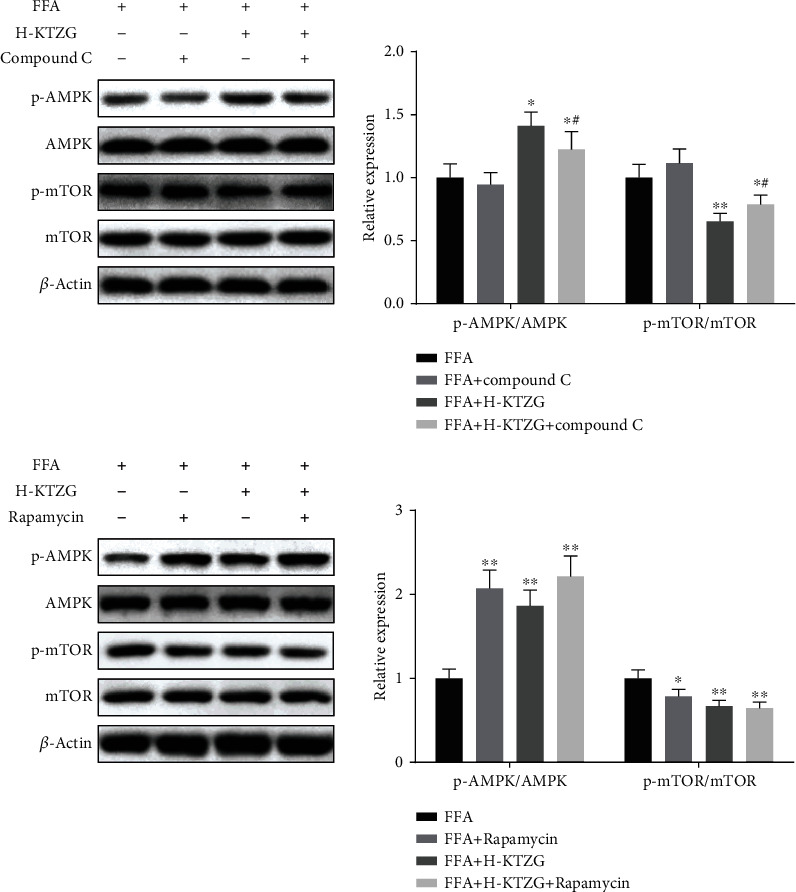
Regulation of KTZG on AMPK/mTOR signaling pathway in FFA-induced HepG2 cells. Compared to FFA group, ^★^*P* < 0.05, ^★★^*P* < 0.01; compared to FFA+compound C group, ^#^*P* < 0.05, ^##^*P* < 0.01.

Compared to the control group, the p-AMPK expression was significantly decreased in FFA-induced HepG2 cells (*P* < 0.01), and KTZG treatment increased the expression significantly in the H-KTZG group (*P* < 0.01); the p-mTOR expression was significantly increased in FFA-induced HepG2 cells (*P* < 0.01), and KTZG treatment increased the expression significantly in the M-KTZG and H-KTZG groups (*P* < 0.01) (Figure 7(b)). Immunofluorescence staining also showed that compared to the control group HepG2 cells, p-AMPK expression was decreased in FFA-induced HepG2 cells significantly (*P* < 0.01) but increased in the KTZG-treated groups significantly (*P* < 0.05 or *P* < 0.01) (Figure 8(a)). p-mTOR was increased in FFA-induced HepG2 cells (*P* < 0.01) but suppressed with KTZG treatment (*P* < 0.05 or *P* < 0.01) (Figure 8(b)). FFA-induced HepG2 cells were further treated with AMPK inhibitor compound C (10 *μ*L) or mTOR inhibitor rapamycin (10 *μ*L) for 24 h. Western blot results showed that compared to FFA+compound C group cells, the protein expressions of p-AMPK in FFA+KTZG+compound C group cells were significantly increased (*P* < 0.05), and the protein expressions of p-mTOR in FFA+KTZG+compound C group cells were significantly suppressed (*P* < 0.05) (Figure 9).

## 4. Discussion

NAFLD is defined as excessive hepatic lipid accumulation in the form of lipid droplets, also known as hepatic steatosis, in the liver with minor or no alcohol intake. It encompasses a broad spectrum of hepatic damage stages such as isolated hepatic steatosis, NASH, liver fibrosis, and cirrhosis or may even progress to hepatocellular carcinoma [12]. In the current study, we explored the effect and underlying mechanism of KTZG on NAFLD with *in vivo* and *in vitro* experiments. As the results indicated, in HFD-fed rats, KTZG treatment significantly improved liver organ index and serum lipid profiles and alleviated hepatic steatosis and liver lipid accumulation; in FFA-treated HepG2 cells, KTZG treatment significantly decreased intracellular TG levels, lipid accumulation, and attenuated glucose uptake. In addition, network pharmacology and experiment study showed that this protective effect of KTZG from hepatic steatosis might be associated with its ability to regulate the AMPK/mTOR signaling pathway.

Excess FFA are converted to TG and stored in hepatocytes, and excessive accumulation of TG in hepatocytes is the pathologic hallmark of NAFLD. The treatment strategies are also generally aimed at reducing TG accumulation. Other lipid profiles, including TC and LDL-C, are also accumulated in hepatocytes with NAFLD. TG/HDL-C and TC/HDL-C ratios are associated with the severity of NAFLD, and the patients with higher lipid ratios had a significantly greater risk for advanced NAFLD [13, 14]. In this study, aberrant lipid profiles were emerged in SD rats fed with HFD; the serum TG, TC, and LDL-C levels were significantly increased; and TG/HDL-C and TC/HDL-C ratios were higher than normal rats; TG levels in HepG2 cells treated with FFA were also increased significantly. In addition, lipid droplet accumulation and liver pathologic damage-like hepatic steatosis were observed in the liver tissues and HepG2 cells with oil red O staining. KTZG treatment restored these altered lipid profiles in HFD rats and HepG2 cells; with the TG, TC, and LDL-C levels decreased, lipid accumulation and hepatic steatosis were also ameliorated significantly.

Although studies focused on this Chinese medicine compound prescription is limited, there are still some studies that could be found for the eight TCM of KTZG against NAFLD. In HepG2 cells, the water extract of *Puerariae radix* treatment could attenuate the hepatic lipoprotein production and secretion; the intracellular total and free TC concentrations also decreased [15]. Metabolomic analysis also revealed that by improving metabolism disorders and inhibiting oxidative damage, the total isoflavones from *Radix Puerariae* could exhibit the therapeutic potential in diabetic rats [16]. In high-fat diet fed with Rhizoma Dioscoreae Tokoronis extracts, mice were found to have lower increases in body and epididymal adipose tissue weights, a lessened occurrence of hepatic steatosis, and a significant decrease in TG, TC, and LDL-C than mice that were fed with high-fat diet only [17]. The mulberry and mulberry leaves are used in TCM as the remedy for obesity, hyperlipidemia, and metabolic disorders [18]. Mulberry leaf extracts could attenuate dyslipidemia and lipid accumulation in obesity-related NAFLD mice, via downregulating the lipogenesis enzymes, inflammation, and oxidative stress while upregulating the lipolysis markers [19]. Mulberry fruit extract could ameliorate lipid accumulation and inhibit the increased levels of TC, TG, and LDL-C but restore the level of HDL-C in HFD-fed rats and protect liver tissue against NAFLD damage through the inhibition of mitochondrial oxidative stress [20].

Network pharmacology and HPLC analysis also clarified the major chemical components of KTZG. Some chemicals, including puerarin, rutin, daidzein, and quercetin, were identified from KTZG using both network pharmacology and HPLC analysis. Wang et al. [21] found that puerarin mediated activation of the PARP-1/PI3K/AKT pathway, and further improvement in fatty acid metabolism could ameliorate NAFLD in C57BL/6J mice fed with a high-fat high-sucrose diet. In addition, the expressions of genes involved in hepatic fatty acid synthesis including PPAR-*α*, SREBP-1, and FAS were also attenuated with puerarin coadministration. In both fat-challenged murine liver tissues and HepG2 cells, rutin treatment was shown to significantly lower TC content and the abundance of lipid droplets and also was able to restore the expression of lipid-related genes, PPAR-*α*, and downstream targets, CPT-1 and CPT-2, while suppressing those of SREBP-1c, DGAT-1, and DGAT-2, as well as ACC [22]. Daidzein was reported to act like a PPAR-*γ* activator, stimulating the adipogenic differentiation in 3T3-L1 adipocytes, and in obese mice, daidzein could inhibit hypertrophy in fat cell size and improved insulin sensitivity, concomitant with upregulation of PPAR-*γ* in fat tissue [23]. Results of *in vivo* and *in vitro* T2DM-induced NAFLD and quercetin treatment models revealed that quercetin could alleviate the serum transaminase and IL-1*β*, IL-6, and TNF-*α* levels; recover oxidative stress; and markedly reduce T2DM-induced histological alterations and lipid accumulation of the livers, accompanied by the restoration of the increased serum total bile acid and the decreased liver total bile acid [24]. These studies indicated that the potential therapeutic effect of NAFLD against HFD-induced lipid accumulation and liver steatosis of NAFLD may owe to these identified chemicals from KTZG.

Based on the identified chemicals in KTZG, some lipid-related genes targeted to these chemicals, including PPAR-*γ*, SREBP-1, FAS, and SIRT1, were also identified with network pharmacology. PPAR-*γ* is critically implicated in the metabolic regulation of lipid and lipoprotein levels, such as TG, and blood glucose. Some drugs like fibrates and glitazones, with the ability to deduce TG levels in NAFLD treatment, are PPAR agonists; they act through the activation of nuclear receptors of the PPAR family, thereby regulating genes involved in TG metabolism [25, 26]. FAS is an important enzyme for fatty acids in controlling lipid synthesis, and SREBP-1 could regulate the expression of FAS to increase lipid synthesis in the liver or adipose tissues [27]. Moderate Sirt1 overexpression protects mice from developing NAFLD, and sirt1-deficient mice witness increased body weight and triggered hepatic steatosis [28]. In HFD-fed rats and FFA-induced HepG2 cells, except for the alleviation effect of KTZG on lipid accumulation and hepatic steatosis, our results showed that KTZG could regulate the expression of these lipid-related genes, upregulating PPAR-*γ* and Sirt and downregulating FAS and SREBP-1. These indicated that KTZG exhibit the therapeutic potential against NAFLD which might be via the active chemicals targeting these lipid-related genes, inhibiting the activity of transcription factors for lipid synthesis, thus inhibiting lipid accumulation. The puerarin identified from KTZG was reported to decrease the expression of lipogenic enzymes, FAS and SREBPs, and activate the AMPK signaling pathway to exert a regulatory effect on lipid accumulation [29]. A study focused on rutin found that in both HFD-challenged mouse liver tissues and HepG2 cells, rutin treatment significantly lowered TG content and the abundance of lipid droplets; furthermore, rutin treatment was able to restore the expression of PPAR-*α* and AMPK, while suppressing the expression of lipid-related genes, SREBP-1 and FAS [22, 30]. Daidzein and quercetin are also reported to have the potential to alleviate hepatic steatosis and lower lipid accumulation; daidzein promotes the expression of fatty acid oxidation- and oxidative phosphorylation-related genes via an ERR*α* pathway to decrease lipid accumulation [24, 31].

The AMPK/mTOR signaling pathway is a master regulator of metabolism and critically involved in metabolic diseases. Activation of AMPK could inhibit the synthesis of fats and protect against diet-induced NAFLD; mTOR is an important signal molecule downstream of AMPK, which also plays a central role in autophagy [32]. In a genetically engineered mouse model, liver-specific AMPK activation reprograms lipid metabolism, reduces liver steatosis, decreases expression of inflammation and fibrosis genes, and leads to significant therapeutic benefits in the context of diet-induced obesity [33]. Other activators of AMPK also have the capability of inhibiting lipid and TC synthesis pathways, lowering hepatic and systemic lipid and TC levels, and these effects require AMPK activity in the hepatocytes [34]. The protective effect of the AMPK/mTOR signaling pathway against NAFLD is also associated with the regulation of autophagy in liver cells. In this study, the expression of p-AMPK was significantly decreased in HFD-fed rats and FFA-induced HepG2 cells; p-mTOR was significantly increased in HFD-fed rats and FFA-induced HepG2 cells. KTZG treatment activated the expression of p-AMPK and suppressed the expression of p-mTOR in liver tissues and hepatocytes, indicating that the AMPK/mTOR signaling pathway may participate in the protective mechanisms of KTZG against NAFLD. Furthermore, compound C, an AMPK inhibitor, and rapamycin, a mTOR inhibitor, were further used to confirm the role of the AMPK/mTOR signaling pathway in KTZG for NAFLD treatment.

## 5. Conclusion

In conclusion, the present study demonstrated that KYZG had a protective effect against NAFLD; its treatment significantly improved the liver organ index and serum lipid profiles and alleviated hepatic steatosis and liver lipid accumulation in HFD diet-fed rats; in FFA-treated HepG2 cells, KTZG treatment also decreased intracellular TG levels and lipid accumulation and attenuated glucose uptake significantly. In addition, these protective mechanisms of KTZG might be associated with its ability to regulate the AMPK/mTOR signaling pathway.

## Data Availability

The data used to support the findings of this study are included within the article.
